# Editorial: Assessment and management of addictive disorders in sexual and racial minorities

**DOI:** 10.3389/fpsyt.2026.1839022

**Published:** 2026-04-15

**Authors:** Liezille Jacobs, Julian Jacobs, Yi-lang Tang

**Affiliations:** 1 Department of Psychology and School of Journalism and Media Studies, Rhodes University, Makhanda, South Africa; 2 Department of Psychiatry and Behavioral Health, Emory University & Atlanta VA Health Care System, Decatur, Georgia

**Keywords:** addictive disorders, behavioral addictions, chemsex, cultural psychiatry, racial minorities, resilience, sexual minorities

## Introduction

Addictive disorders rarely emerge in isolation. They are deeply entangled with biological, psychological, and the social factors. Sexual and racial minorities face a heightened risk of developing substance use disorders (SUDs) and addictive disorders compared to heterosexual and non-minority peers. This risk is particularly pronounced among individuals with intersecting marginalized identities, such as lesbian, gay, and bisexual (LGB) adults in racial minority groups, who demonstrate higher odds of SUD and co-occurring suicidal ideation relative to non-minority heterosexuals (1, PMID: 34214885; 2, PMID: 33821743) (3, PMID: 40536205). For these populations, the complexity of addiction is further complicated by stigma, discrimination, and inequities in access to care. Minority stress, including discrimination and adverse childhood and adult experiences, is a key mediator of these disparities. Sexual minorities report greater exposure to trauma and discrimination, which in turn increases their vulnerability to both SUD and comorbid mental health disorders (4, PMID: 33264820; 5, PMID: 31761516).

These intersecting forces not only shape vulnerability to addiction but also influence how individuals seek help, engage with treatment, and experience recovery. This Research Topic of Frontiers in Psychiatry invites readers to look beyond narrow, conventional framings and to engage with new ways of understanding, assessing, and managing addictive disorders. Across six contributions spanning continents, populations, and methods, the issue underscores that the future of addiction psychiatry must be intersectional, inclusive, and attentive to lived experience.

## Innovation in treatment: neurobiology meets psychotherapy

In Indonesia, Siste et al., in “Utilizing repetitive transcranial magnetic stimulation in the management of gambling disorder in Indonesia: protocol for a pilot and feasibility study”, introduce a pilot protocol that combines repetitive transcranial magnetic stimulation (rTMS) with cognitive-behavioral therapy (CBT). Gambling disorder is recognized in both DSM-5 and ICD-11, yet treatment options remain limited, particularly in low-resource settings.

The study’s significance lies in its exploration of a multimodal approach for a behavioral addiction with few established pharmacological options. By targeting both cognitive distortions and neurobiological dysregulation, the combined rTMS-CBT model may enhance treatment adherence, symptom reduction, and relapse prevention. If feasible and effective, the protocol could serve as a basis for broader clinical adoption in Indonesia and comparable contexts where treatment infrastructure is scarce.

## Conceptual frameworks: rethinking sexualized drug use

In “Polydrug use during chemsex: single and intersecting sexual effects of commonly used drugs”, Platteau et al. propose a layered, visual model to capture the complexity of chemsex. Instead of focusing narrowly on a few substances, the Pharmacosex Wheel maps drugs according to both pharmacological properties and their sexual effects. The Layered Model goes further by accounting for intersecting drug effects when substances are combined, thereby recognizing the poly-drug practices common in chemsex settings. Importantly, the authors highlight that motivations for sexualized drug use extend beyond simple pleasure-seeking to include coping with stigma, enhancing intimacy, or negotiating sexual identity, particularly among gay, bisexual, and other men who have sex with men (MSM). By foregrounding motivations ranging from pleasure and intimacy to coping with stigma, the framework challenges one-size-fits-all approaches and opens the door to more tailored, context-sensitive harm-reduction strategies.

## Intersecting vulnerabilities: sleep, substance use, and sexual risk

Sari et al., in their study “Poor sleep quality indirectly contributes to higher sexual risk-taking by increasing the likelihood of engaging in substance use among LGBTQ+ individuals” highlights how vulnerabilities compound in Turkey, where over 80% of participants reported poor sleep quality. It addresses an underexplored area, as much of the research on sexual health and substance use has historically focused on men who have sex with men (MSM), leaving women and gender-diverse individuals understudied. Poor sleep indirectly increased sexual risk-taking, primarily through heightened use of chemsex-related drugs. This underscores sleep as a neglected but crucial dimension of addiction care and situates addiction within a sociopolitical context of minority stress.

## Chemsex as both risk and meaning

Mundy et al. (6), in “The complex social, cultural and psychological drivers of the ‘chemsex’ experiences of men who have sex with men: a systematic review and conceptual thematic synthesis of qualitative studies”, synthesize 43 qualitative studies to show that chemsex carries both dangers and meanings. The review examines the complex motivations, contexts, and consequences of chemsex, highlighting its social, cultural, and psychological underpinnings.

While associated with HIV transmission, overdoses, and psychological distress, chemsex was also experienced as a space of intimacy, community, sexual exploration, and belonging (identity construction). Their review calls for culturally sensitive and non-judgmental interventions that balance harm reduction with respect for agency and identity.

## Beyond chemsex: behavioral addictions and resilience

In the study of Zhang et al., “Network analysis of interactions of rumination and anxiety on smartphone dependence symptoms”, the authors surveyed Chinese preservice teachers (N = 1,610) using validated instruments: the Ruminative Response Scale-10, Generalized Anxiety Disorder Scale-7, and Mobile Phone Addiction Index-17. Through network analysis, the authors identified key “bridge nodes” that connect rumination and anxiety to symptoms of addictive smartphone use in East Asia.

Importantly, the study identified key “bridge nodes” connecting susceptibility factors (rumination and anxiety) to Smart Phone Dependence (SPD) symptoms. Among these, the reflection-oriented behavior of “writing down thoughts and analyzing them” (R5) emerged as the most central and influential node. It connected strongly to both uncontrollable craving symptoms (e.g., hiding phone use, overspending on bills) and to anxiety-driven symptoms of feeling lost or disconnected without a phone. This suggests that certain reflective forms of rumination, while sometimes adaptive, may exacerbate SPD when paired with anxiety.

## Prevalence and patterns of substance use among clients utilizing Nova Scotia’s centralized intake for mental health and addiction services

Soboka et al. conducted a large cross-sectional study (N = 22,500) analyzing the substance use profiles of adults contacting Nova Scotia’s centralized Mental Health and Addictions (MHA) intake service (2020–2021). The study revealed a high burden of use, with 36.1% of clients reporting daily substance use. This rate surged to nearly 70% among individuals experiencing homelessness.

While alcohol, tobacco, and cannabis were the most common substances overall, clients reporting use of specific drugs daily showed alarming rates of opioid (69.0%) and polysubstance (44.4%) use, primarily administered via smoking and injection.

Multinomial analysis identified significant associations between daily use and several vulnerabilities. Men and non-binary individuals were more likely to use substances across all frequencies than women. Daily use was also strongly linked to being young (19–29), having multiple psychosocial stressors, lacking private insurance, having multiple co-occurring illnesses, and being at high suicide risk.

The authors conclude that substance use is driven by the intersection of race, gender, socioeconomic disadvantage, and mental health burden. The findings highlight the critical need for integrated care models and structural interventions that address underlying determinants like poverty and housing instability to reduce the high prevalence of daily and polysubstance use in this vulnerable population.

## Looking ahead: toward inclusive addiction psychiatry

Across these contributions, several common threads emerge. Effective interventions must be culturally grounded and contextually adapted. Innovative frameworks such as the Pharmacosex Wheel and network analysis provide new ways of understanding complexity and tailoring care. Addiction psychiatry must move beyond pathologizing narratives to embrace both risks and meanings, particularly for sexual minorities.

As readers engage with this Research Topic, we invite reflection on pressing questions: How can psychiatry balance acknowledgement of harm with respect for agency and pleasure?

What role might sleep health and resilience-building play in prevention and treatment? And how can we design care systems that not only reduce symptoms but also affirm identities and foster belonging for sexual and racial minorities?

By weaving together innovations in neurobiology, conceptual clarity, and sociocultural nuance, these articles point toward a new approach that is intersectional, inclusive, and deeply responsive to lived experience. Our effort is only the beginning. Future research must expand the scope of inquiry, including the use of emerging technologies to reach and support marginalized populations. There is also a pressing need for more interventional studies, particularly longitudinal research, to evaluate how diverse, identity-affirming interventions influence long-term recovery trajectories and mental health outcomes.
